# What matters in development and sustainment of community dementia friendly initiatives and why? A realist multiple case study

**DOI:** 10.1186/s12889-023-15125-9

**Published:** 2023-02-09

**Authors:** Marjolein Thijssen, Wietske Kuijer-Siebelink, Monique A.S. Lexis, Maria W. G. Nijhuis-van der Sanden, Ramon Daniels, Maud Graff

**Affiliations:** 1 Radboud University Medical Center, Radboud Institute for Health Sciences, Scientific Center for Quality of Healthcare (IQ healthcare), Nijmegen, The Netherlands; 2 Radboud University Medical Center, Radboud Alzheimer Center, Nijmegen, The Netherlands; 3 HAN University of Applied Sciences, School of Education, Nijmegen, The Netherlands; 4 Radboud University Medical Center, Radboudumc Health Academy, Research on Learning and Education, Nijmegen, the Netherlands; 5 Zuyd University of Applied Sciences, Research Centre Assistive Technology in Care, Heerlen, the Netherlands

**Keywords:** Dementia, Dementia friendly communities, Dementia friendly initiatives, Community, Realist approach, Social participation, Levels of change development, Sustainment

## Abstract

**Background:**

Dementia friendly communities (DFCs) are seen as key to participation of people with dementia and carers. Dementia-friendly initiatives (DFI) are important building blocks for the growth of DFCs. Therefore, it is essential to understand how DFIs are developed and sustained to secure the growth of DFCs. This study identifies contextual factors and mechanisms that influence the development and sustainment of Dutch DFIs. It also explains how these contextual factors and mechanisms are interrelated and the outcomes to which they lead.

**Methods:**

Mixed methods, namely interviews, observations, documentation and focus groups, were used for this realist multiple case study. Participants were professionals (*n* = 46), volunteers (*n* = 20), people with dementia (*n* = 1) and carers (*n* = 2) who were involved in development and sustainment of DFIs in four Dutch DFCs.

**Results:**

This study revealed three middle-range program theories as final outcomes: development of a support base, collaboration, and participation in DFIs by people with dementia and carers. These theories address institutional, organisational, interpersonal and individual levels in the community that are essential in development and sustainment of DFIs.

**Conclusions:**

The development and sustainment of DFIs requires the development of a support base, collaboration, and participation in DFIs by people with dementia and their carers.

**Supplementary Information:**

The online version contains supplementary material available at 10.1186/s12889-023-15125-9.

## Background

Growing recognition of dementia as an urgent global health issue has led to an increase in dementia-friendly communities [1]. Dementia-friendly communities (DFCs) share a common goal of ensuring that people with dementia and their carers continue to participate and be valued as citizens [2–4]. DFCs can be characterised by their location, such as a city or neighbourhood. These are called ‘location-based DFCs’ [5, 6]. DFCs can also be organisations or entities with a specific focus (for example, airports). These are referred as ‘communities of interests’ [5, 6]. The need for building DFCs is recognised by 90 % of Organization for Economic Co-operation and Development (OECD) countries, including the Netherlands. The need and ambition to build Dutch DFC is captured in a national strategy called Deltaplan Dementie [7, 8].

Although this need to nurture DFCs is widely recognised, the development of DFCs is influenced by a broad range of stakeholders and organisational factors that make this task complex. Previous research has shown that DFCs require both top-down input by the (local) government, such as policy, facilitation and finances, and bottom-up (local) resources and initiatives, such as initiatives focusing on awareness about dementia and related social interaction [3, 6, 9, 10]. Such dementia-friendly initiatives (DFIs) are the ‘building blocks’ in the development of DFCs [6, 11]. DFIs are initiatives and activities that aim to promote dignity, empowerment, engagement and autonomy to enhance the wellbeing of people with dementia and their carers, and to address the needs of carers throughout the dementia trajectory [11]. The specific goal of DFIs is to bring about changes to the social and/or physical environment to create DFC. DFIs therefore work towards a community that includes and empowers people with dementia and their carers. The term, “dementia-friendly community” refers to the kind of community to strive for, where everyone, including people with dementia, has a place. By “dementiafriendly initiatives” we mean the activities being undertaken to make communities more inclusive of people with dementia and their carers [12]. As such, a DFC can evolve from a collection of DFIs [6, 13]. For example, a neighbourhood DFC can have different DFIs, such as education about dementia and related social interaction for supermarkets and/or adaptations to the physical environment to improve recognition in that environment.

In the Netherlands, decentralisation of health and care policies and services makes municipalities responsible for providing appropriate dementia care and support, as stated by the Social Support Act 2015 [14–16] Therefore, building DFC is a responsibility of municipalities [7, 8]. This has automatically led to an increase of local DFIs [7, 16]. Local DFIs may vary significantly in format, structure, content and outcomes, based on local resources and needs, the partners engaged and population groups targeted [7, 12, 17, 18]. That means that one municipality decides to initiate a Meeting Centre as a DFI while another municipality focuses on integrating dementia in existing activities such as a choir or community garden.

The development of DFIs is a complex local process. It requires commitment of key actors in a local context, ranging from municipalities and healthcare and social organisations to businesses and voluntary-sector organisations [3, 8, 19, 20]. They operate within the local processes and structures of municipal, health and social systems, with their resources and restrictions [3, 19]. Because of the local nature of the development of Dutch DFIs, more contextual in depth knowledge is needed how to develop and sustain DFIs, which previous research about DFCs have not explicated [6, 21, 22]. No previous research has investigated how key people from different backgrounds come and work together to develop and sustain a DFI. No research has clarified which contextual factors are critical to set up a DFI, for whom they are needed, and how these factors add to the development and sustainment of a DFI. Clearly, more in depth knowledge is needed about how DFIs are developed and sustained in order to secure the evolution of DFCs.

The aim of this study was to identify contextual factors and mechanisms that influence the development and sustainment of Dutch DFIs while building a DFC, and explain how they are interrelated. To this end, a multiple case study was carried out using a realist approach. The main goal of the realist approach is to illuminate how complex social interventions work, using context-mechanisms-outcome configurations from one or more realist program theories, to answer the questions: ‘what works, for whom, in what contexts and why’ by describing causal relationships to explain outcomes [23, 24]. The focus of the realist approach on identifying mechanisms and exploring how they operate in different contexts also provides valuable insights into how and why intervention programmes lead to change. The realist approach can add value by enhancing the clarity, depth, and portability of findings, helping professionals and researchers deal with context and complexity in pragmatic ways [25–27]. .Furthermore, the realist approach enables exploration of causal processes within and across multiple levels of a social system, as occurs in the development and sustainment of DFIs [25, 28, 29]. These characterizations of the realist approach are particularly useful when (future) DFC wish to learn how to apply lessons from a local DFC elsewhere.

The research question(s) were:

- Which mechanisms are important in developing and sustaining DFIs, what outcomes do they have, and why?
- Which contextual aspects affect these mechanisms?

This research was the second phase of the Mentality Project (November 2017–October 2022), which studies success factors in DFIs using the realist approach. The research was guided by the research team and an advisory panel consisting of experts in the field of dementia and public health, representatives of people with dementia and their caregivers and stakeholders from four Dutch municipalities seeking to become dementia-friendly. More information about Mentality can be found at www.Mentality.space.

## Methods

A multiple case study design using the realist approach was chosen because it allowed in-depth study of development and sustainment of DFIs within the real-life context of Dutch location-based DFCs [30]. Multiple methods of data collection were performed, including semi-structured interviews, participating observations, available documents and focus groups.

### Case selection

Selected cases were DFCs that were officially recognised as such according to Dutch Alzheimer Association’s criteria [16]. Within the cases of Dutch DFCs, a DFI was our unit of analyses, which means that the DFI was the case study topic to be studied [30]. Purposeful sampling, as a method to select information rich cases [31, 32] was used based on maximum variation of DFCs in terms of rurality (urban vs. rural sites), the geographic scatter in the Netherlands, duration of being a DFC and characteristics of DFIs within a DFC [10]. This sequential sampling method was established together with the advisory panel of Mentality. A multiple recruitment strategy was adopted by identifying cases through the cooperation of the Dutch Alzheimer’s association, the advisory panel of Mentality, and the network of the research team at national and/or regional level.

Twenty-five potential DFCs were identified and were invited by the research coordinator (JP) to take part. Nine DFCs were willing to participate, and were considered in terms of the maximum variation and the opportunity for identifying contextual factors, mechanisms and outcomes. Four DFCs were selected by the research team and advisory panel as best possible sites.

After the initial selection of DFCs, additional information was sent to key informants, such as policy officers. The key informants informed and consulted other stakeholders involved in the development and sustainment of DFIs, such as social and health professionals and/or volunteers, before making definite commitments. After this step, a letter of commitment was signed by the local policy officer for each case.

Table 1 provides an overview of included cases and their characteristics.

**Table 1:** Included Cases and Their Characteristics

DFC	A.	B.	C.	D.
Selection criteria				
DFC since	2015	2019	2018	2015
Urban vs rural	Rural	Urban	Rural	Urban
National geographic dispersion	South-east	West	North	South
Dementia friendly initiative (DFI)	Inter-generational gardening and adapting the physical environment	Alzheimer Café and Odense housing	Dementia-friendly museum	Dementia-friendly choir and shared living room for older people, including people with dementia

### Recruitment of participants within the DFCs

Recruitment of participants within DFCs was organized around each DFI as unit of analysis. The DFIs were developed to include people with dementia, sometimes in existing activities such as the intergenerational gardening, dementia-friendly museum, dementia-friendly choir and shared living room for older people, including people with dementia and sometimes by specific dementia friendly activities such as adapting the physical environment, Alzheimer Café and Odense housing. Participants were stakeholders who were actively involved in the development and sustainment of one or more DFIs. The aim was to include a variety of stakeholders who were involved at both the municipal and implementation level of one or more DFIs. Given time constraints, we set a target of 7–12 participants per DFC for project manageability.

Participants were recruited through first purposeful sampling [31, 32] by selecting key informants in each DFC and then snowball sampling [32] which means that key informants were asked to identify other information-rich informants who were active and engaged in the development and implementation of DFIs. As such, participants assisted in the identification of other eligible participants [32].

### Data collection

Following the realist and case study methodology, multiple methods of data collection were used. These were semi-structured interviews, participating observations, available documents and focus groups. In all cases, data collection started with semi-structured interviews, interspersed with observations and documentation. Data collection was divided among four researchers. One pair (MT and JP) collected data in A and B. Another pair (ML and RJ) collected data in C and D. All data were collected between January 2019 and July 2019. See Table 2 for an overview of data collection.

**Table 2:** Overview of Participants by Case and Data Collection Method

	A.	B.	C.	D.
**Total Interviews**	**7**	**9**	**12**	**12**
Interviews professionals	5	5	9	10
Interviews volunteers	2	4	2	2
Interviews people with dementia or carers as co-developers	0	0	1	0
**Total Participating observations**	**3**	**5**	**1**	**2**
Activities involving participatory observation	Walking and working in the garden.	lecture and discussion in Alzheimer café, reading paper and playing board games in Odense housing	Presentation and discussion about Egyptian pharaohs	Participation during choir practice
**Total Documents**	**4**	**5**	**5**	**3**
Content of documentation	Proposals for government grants, policy papers	Evaluations, policy papers	Policy papers	Policy papers
**Total Number of Participants in Focus Groups**	**4**	**13**	**7**	**4**
Professionals	2	8	6	1
Volunteers	2	4	1	3
Carers as users of DFIs	0	1	0	1

### Semi-structured interviews

Semi-structured interviews were conducted using a topic guide following the realist principles. They included open-ended and exploratory questions for developing program theories [33]. The purpose was to gain insight into the nature of the DFI; the involvement of the interviewee in the DFI; and their experiences with and perspectives on how the DFI was developed, implemented and sustained. Special attention was given to contextual aspects and mechanisms leading to outcomes. All interviews were audio recorded with the permission of the participant(s). Each interview lasted approximately 40–60 minutes, and took place face to face, on location, chosen by the participants. These locations were mostly at their homes or at work. In total, 29 professionals, 10 volunteers and 1 person with dementia were interviewed. The topic guide is available in Additional File 1.

#### Participating observations

The purpose of the participating observations was to gain insight into the implementation of DFI and to collect information regarding the context, such as the social and physical environment. Open notes were made during and after the observation sessions, and were used for probing contexts, mechanisms or outcomes during interviews and focus groups. Each observation lasted approximately 40–60 minutes and took place at the location of the DFI. In total, 11 participating observations of DFI were performed.

#### Documentation

Documents related to organisational policy papers, evaluations of and proposals for DFIs were studied. These documents, collected and delivered by the interview participants, aimed to contextualise and supplement data from interviews and observations. In total, 17 documents were collected.

#### Focus groups

In each case, interim findings from the interviews, observations and documents were presented as summary reports during a focus group meeting with the participants from interviews and observations, as well as other relevant participants who could not be interviewed individually. This was a basis for member checking, discussion to highlight unanswered questions and deepen the findings for theory development. Each focus group lasted approximately 60–90 minutes and took place at a location, chosen by the participants. These locations were at the community centre (case A) and city hall (case B,C and D). In total, 17 professionals, 10 volunteers and 2 carers participated in the focus groups. Table 2 shows the overview of participants, specified per case and data collection method.

### Data extraction

Data extraction started per case after data from interviews, participating observations and documentation were collected. To ensure consistency and transparency, definitions of ‘contexts’, ‘mechanism resources’, ‘mechanism response’ and ‘outcomes’ were used [27, 34–36]. See Table 3 for definitions.

**Table 3:** Definitions for Data Extraction

**Context** pertains to the backdrop of an intervention [37]. Context includes the pre-existing organisational structures; the cultural norms and history of the community; the nature and scope of pre-existing networks; and geographic location effects, such as social and physical environment or previous experience with dementia-friendly initiatives [37]. **Mechanisms** are not interventions. They are the – often invisible – forces, powers, processes or interactions that lead to (or inhibit) change. They can be found in the choices, reasoning and decisions that people make as a result of the resources; the interactions between individuals or groups; and the powers and liabilities that things, people or institutions have as a result of their position in a group or society [38]. Mechanisms are ‘triggered’ when (program) resources (e.g. information, money, expertise) interact with specific features of the context (individual, interpersonal, organizational, or institutional) [25]. *Mechanisms resources* refer to what is triggered among the context of participants/stakeholders [36, 37]. *Mechanisms response* refers to the responses of the participants, all that suggests a change in people’s minds and actions [36, 37]. **Outcomes** are the results of how people react to the mechanisms. Outcomes are either intended or unintended and can be proximal, intermediate and final [26, 27]. **Outcomes can be labelled as:** • observed (the participant stated during interviews or observations that it had happened); • anticipated (it had not happened yet but the participant expected it to); or • implied (no explicit mention of the outcome was made but the data enabled the research team to infer, tentatively, that the participant had observed or anticipated it) [27]. **Labelling levels of change in mechanisms:** • Individual changes include individuals’ skills and knowledge relating to dementia and DFIs, as well as the motivation, attitudes, commitment and values that affect individual behaviour [27]. • Interpersonal and network change refers to the relationships and networks between individuals and groups that influence development or sustainability of DFIs [27]. • Organisational change refers to the systems, policies and procedures, practices, culture and norms within an organisation that affect the access and resources needed to develop and sustain DFIs [27]. • Institutional change relates to the wider environment within which individuals, networks and organisations operate. This includes the political system, civil society and the media, political and economic factors, and broader social factors (culture, norms, collective beliefs) that influence the development and sustainability of DFIs [27].	

Following the realist approach, the researchers (MT and AOB) constructed context-mechanism-outcome configurations (CMOc) from each interview transcript in order to examine what worked for whom, under what circumstances, and why and how [28]. Interviews were thus analysed using CMOc, which were drafted and placed (by AOB/ MT) into a data extraction form in Excel, which is available in Additional File 2. Next, data from field notes and documents was extracted and supplemented to the CMOc. Corresponding CMOc were triangulated, noting the data source and corresponding fieldnotes or documentation. If new or rival data were found, they were extracted in new CMOc.

Labelling CMOC on outcomes and levels of change.

In the last steps of data extraction, outcomes of each CMOc were labelled according to whether they were observed, anticipated or implied by the data source, following the reasoning that any insight into relevant contextual factors or mechanisms must relate to an outcome [27]. Each CMOc was also labelled on levels of change as defined by Punton [27] to deepen the data-extraction on mechanisms, since change occurs at different social strata, consistent with the development of DFI [27, 37, 39]. Thus, researchers (AOB/MT) labelled each CMOC according to the level on which change took place – the individual, interpersonal, organisational or institutional [27].

### Data synthesis

Data synthesis used an inductive, sequential and iterative approach [30, 40]. Sythesis of information within each case was followed by a cross-case synthesis and finally overall synthesis. All authors participated in each step.

#### Within-case synthesis

For this, we used retroduction, which is a central inference-making method in realist research [41]. Retroductive theorizing involves starting with a program’s effects and working backward to think about the conditions of reality necessary for such effects to manifest [27, 42, 43] Within case synthesis started by identifying concrete outcomes (‘what were changes so that DFIs were developed and sustained?’) and then working backwards to mechanisms (‘how and why did these changes occur?’) and then identifying contexts (‘under what circumstances will these mechanisms lead to these changes?’) [27, 42–44]. As such, similar outcomes, such as an improved perception of the importance of a DFI or taking initiative, were clustered per case. Second, commonalities of mechanisms and contexts were also clustered, such as mechanisms referring to feeling important or contextual aspects such as stakeholders from various organizations. Third, based on the clusters, patterns in outcomes, mechanisms and contexts were outlined. An example of such an outline was contextual factors, such as stakeholders having a personal affinity with people with dementia and carers, leading to mechanisms such as feelings of being important or connected, which then themselves led to outcomes such as taking initiative or purposefulness in collaboration. The levels on which change occurred remained distinct. These outlines were compared with corresponding configurations and quotes from data sources, to check for consistency and explanatory power [27, 44].

#### Cross-case synthesis

Cross case synthesis started by following the same three steps as within-case synthesis, namely clustering of the outcomes, followed by clustering of commonalities of mechanisms and contexts, and finally developing outlines. This resulted in ten outlines, including intermediate outcomes in which mechanisms remained distinct on levels of change. The ten outlines are available in Additional File 3. These outlines, including the accompanying levels of change, were discussed with all authors. This discussion confirmed the synthesis and made suggestions for wording.

#### Configuring middle-range program theories

Next, the ten outlines were configurated into initial middle-range program theories (MRPTs) by clustering outcomes and selecting the most prevalent mechanisms, including their contextual factors, by identifying demi-regularities through retroductive reasoning [27, 42–44] This was carried out by three researchers independently (MT, WK and LD) and overlap and differences were discussed until consensus was reached. After this step, the initial MRPTs were presented to all authors and the advisory panel for their feedback. They discussed the MRPTs using their field expertise and confirmed those MRPTs’ usefulness and applicability. Suggestions for wording led to the final MRPTs.

Formulating realist program theories at a midrange level, such as middle-range program theories (MRPT), enabled both the specification of contexts, resources responses leading to outcomes and the conceptualization and explanation of those outcomes [45]. Such MRPTs constituted more granular hypotheses about specific causal links and processes to communicate the findings as concretely as possible in practice compared to the more abstract middle-range theories [25].

## Results

The following section describes three final MRPTs. Each MRPT is represented by a logic model complemented by a narrative as this allows a description of essential features of both actions and change [17, 46]. Each MRPT describes a final outcome in the title and explains how these outcomes are built by contextual factors, mechanisms and lead to intermediate outcomes. Special attention is given to the contextual factors, resources and the levels of change.

### MRPT 1: development of baseline support for a dementia friendly initiative (DFI)

**Fig. 1:**
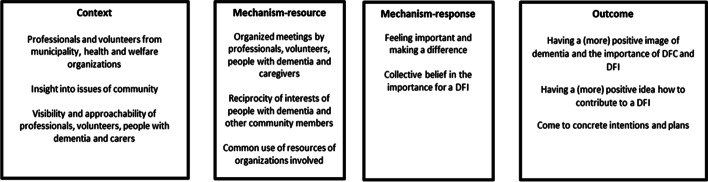
Middle-range Program Theory: Development of a Support Base

This section describes the MRPT of the development of baseline support for a dementia friendly initiative. Figure 1 outlines this MRPT.

For development of baseline support for DFIs, professionals and volunteers from municipalities and health and welfare organisations had insight in current issues in the community. These included needs related to dementia and/or from people with dementia and their carers. Other contextual features were the visibility and approachability of the professionals, volunteers, persons with dementia and carers, so that community members knew their background and could more easily approach them, for example to ask questions. Actions such as organising information meetings about dementia or making plans for a DFI for community members were conducted by the professionals and volunteers sequentially, preferably together with people with dementia and carers. During those actions, resources from organizations were commonly used, such as the physical location of an organization or PR supplies from an Alzheimer’s association. Those actions addressed the interests of both people with dementia and their carers as well as other community members, such as parents and their children, leading to a reciprocity of interests. On the organizational level, feelings of importance and making a difference arose among professionals and volunteers, because they felt that the interest of their organisations or background resonated with interest of other stakeholders and that their resources, such as a physical location or network, were important for a mutual interest, such as a DFI. On the institutional level, community members from the neighbourhood also felt that their interests resonated with interests of other stakeholders and that their resources, such as time or network, had the same importance for a mutual interest, such as a DFI. Feeling important and making a difference arose through reciprocity of interests and recognition that individual interests also mattered for a greater purpose. Therefore, it resonated with feelings to make a positive difference for others. Such responses changed collective beliefs about needs and possibilities for a DFI and therefore brought about changes on an institutional level. Intermediate outcomes of building support for a DFI were having a (more) positive image of dementia and the importance of DFCs and DFIs, and having a (more) positive idea how to contribute to a DFI and develop concrete intentions and plans.

### MRPT 2: collaboration for developing and sustaining DFIs

**Fig. 2:**
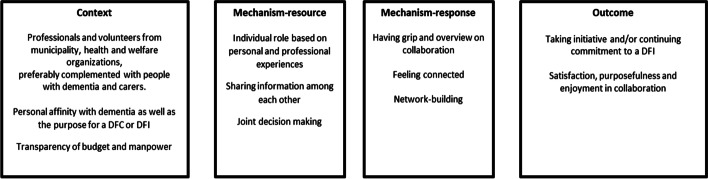
Middle-range Program Theory: Collaboration to Develop and Sustain DFIs

This section describes the MRPT of collaboration for a DFI. Figure 2 outlines the characteristics of this MRPT.

For collaboration, professionals and volunteers from municipalities, health and welfare organisations came together, preferably complemented by people with dementia and their carers. They all shared a personal affinity with dementia or the purpose of a DFC or DFI from various experiences. For example, there was a policy officer who understood the impact of dementia from personal experience as a carer and a volunteer who used to work in elderly care. Other contextual features were transparency about manpower and available budget. As a result, the professionals and volunteers involved knew the preconditions to come together. Follow-up actions were organising regular meetings about the DFIs to be developed or sustained. At these meetings, professionals and volunteers took roles that best suited their personal and professional experiences. During those meetings, relevant information was shared, such as information about history and/or developments in the community or which funds to apply for. Accordingly, meetings were characterised by sharing, and subsequently by joint decision making. These contextual features and resources on the interpersonal level led to responses of both having a grip on and overview of collaboration, and of feeling connected with each other. Such responses led to mutual network building and therefore brought changes on the interpersonal level. Intermediate outcomes of collaboration were taking initiative and/or continuing commitment to a DFI and experiencing purposefulness, as well as satisfaction and fun during the collaboration.

### MRPT 3: participation in DFI by people with dementia and their carers

**Fig. 3:**
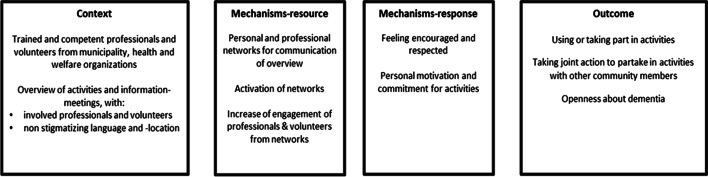
Middle-range Program Theory: Participation in DFIs by People with Dementia and their Carers

This section describes the MRPT of participation in DFIs by people with dementia and their carers. Figure 3 outlines the characteristics of this MRPT.

To enable participation in DFIs by people with dementia and their carers, an overview was developed by dementia trained and competent professionals and/or volunteers from municipalities and health and welfare organizations. This overview outlined the needs of people with dementia and their carers in terms of activities and information meetings, by whom these were delivered and at what locations. To avoid stigmatisation and support inclusion, the overview purposely did not include terms associated with dementia. Therefore, the term ‘dementia-friendly initiative’ was not used. The locations, such as a community garden or a community centre, were also chosen to be inclusive and not associated with dementia. Sequentially, actions were undertaken to communicate this overview. These actions included for example publication in a local newspaper and word of mouth information sharing by professionals and volunteers. During these actions, resources such as the personal and professional networks of professionals and volunteers were used. Moreover, professionals and volunteers from these networks became more engaged in the DFIs and their purpose as they were activated in promoting these initiatives. These contextual features and resources on the interpersonal level led to responses of feelings of encouragement among people with dementia and carers. For example, they identified with activities or information meetings that related to their needs. Another response was that people with dementia and their carers felt respected, for example by recognition of their needs in the overview or by deciding for themselves whether and when they would participate in which activities. Such responses increased personal motivation and commitment to participate, and therefore brought changes on the individual level. Intermediate outcomes of participation in DFIs by people with dementia and their carers were using or taking part in activities and taking joint action to partake in activities with other community members. In doing so, people with dementia and their carers, as well as community members, became more open to the presence of (people with) dementia.

Table 4 shows an overview of the MRPTs and associated levels of change.

**Table 4:** Overview of the Middle-range Program Theories and Associated Levels of Change

Middle Range program theory	Level of Change
Development of a support base	Institutional
	Organisational
Collaboration for developing and sustaining DFIs	Interpersonal
Participation in DFIs by people with dementia and their carers	Individual

## Discussion

### Implications of the findings in context of the existing research

We aimed to explain which mechanisms are important in developing and sustaining DFIs in a Dutch DFC, which outcomes they produced and which contextual factors affected these mechanisms. To our knowledge, this is the first study that has addressed the development and sustainment of DFIs including the different levels on which changes occur. Our analysis revealed three MRPTs in developing and sustaining DFIs. These theories relate to the development of baseline support, collaboration, and participation in DFIs by people with dementia and their carers. Moreover, our results further clarify how each MRPT brought about changes on different levels in the community. Development of baseline support caused changes on the organizational and institutional level, collaboration caused changes on the interpersonal level, and participation in DFIs by people with dementia and their carers caused changes on the interpersonal and individual levels. Our results support previous research about the development and sustainment of complex interventions in dementia community care, which confirms the importance of a support base [5, 10, 12, 47], collaboration by diverse partners [10, 12, 47, 48] and an understanding of pre-requisites for participating in initiatives [10, 47, 48]. Moreover, our results highlight how contextual aspects such as diversity among partners and insight into issues in the community, as well as mechanisms such as sharing of resources and reciprocity of interests, provoke feelings of importance, connection and encouragement among all community members. For example, in our study, the diversity of professionals and volunteers supported the sharing of each other’s resources, such as spaces, information and expertise. Likewise, reciprocity arose through the connection of personal interests with needs and initiatives in the community, and generated significance, engagement and activity. For example, when a neighbourhood was informed about widening the sidewalks and landscaping for people with dementia, the benefits of these initiatives for people with dementia and carers were connected with benefits for parents with strollers and children’s’ play and educational options. Consequently, people became committed to each other and engaged with the purpose of the DFI. Of most interest is that these mechanisms of sharing, reciprocity, significance, connection and engagement rely much more on what connects people together and on positive aspects, rather than what a separate and/or vulnerable group needs. This marks a shift in setting up DFIs in which it is not so much the negative consequences of dementia that act as the impetus for change, but rather a cause that connects residents in the community. Such impetus for change differs from existing toolkits [12, 49–51] and is known as asset based community development (ABCD), an approach which focusses on assets and strengths in individuals and communities, rather than on their problems and deficits [52]. The ABCD approach is used for empowering communities in addressing health inequalities and health promotion in groups from adolescents [53–56] to older people [57–59]. The asset-based approach has recently been promoted as an alternative policy for DFC [59–61]. Moreover, the importance of moving away from deficits and focus on abilities was also highlighted to explain the outcomes of community DFIs for people with dementia and their carers [17]. Our current results explain how a focus on the abilities and wishes of people with dementia, carers and other community members during development and sustainment of DFIs can be the basis for positive responses such as sharing, reciprocity, significance, connection and engagement. As such, our results aligns with the new proposed policy for DFCs and offers new insights into positive responses that are needed for the process in development and sustainment of DFIs. It enables reflection on how and why the process was successful, or not.

Within the context of Dutch DFCs, our results describe how institutional, organizational, interpersonal and individual levels of change are important in the development and sustainment of DFIs. In particular, they explain how each MRPT affects one or two different levels in the community. As such, our results resonate with findings about development of DFCs that address the importance of both top-down and bottom-up approaches [6, 10, 62]. However, our results deepen these findings by focusing and clarifying which contextual aspects and resources are needed and why, and how they trigger mechanisms in different levels of change to build DFIs as building blocks for a DFC. Next, our results suggest an interdependence between the mechanisms, intermediate outcomes of MRPTs and associated levels of change. For example, the positivity from the intermediate outcomes of a support base is likely to generate commitment and willingness to participate in a collaboration. As such, it can start a collaboration or intensify it by moving others to join or spending more effort in it. Consequently, the activity from the intermediate outcomes of collaboration may lead competent professionals and volunteers to support participation in a DFI. Additionally, the positive intermediate outcomes of participation in DFIs, such as openness about dementia and joint participation in activities, can also contribute to a positive idea of a person’s own contribution to a DFI, and thus contribute to a support base. Such examples of interdependencies explain how the intermediate outcomes of one MRPT may become (a part of) the context in a new MRPT [17, 37] without suggesting that there is only one possible direction in which interdependencies can exist. Our results regarding levels of change provide evidence and insights that encourage reflection on the development and sustainment of DFIs.

### Strengths and limitations of the study

Since the development of Dutch DFC and DFIs is locally bound, we included cases from different regions in the Netherland so diversity in contexts could be explored in depth. Additionally, a mixed method approach and a large number of participants (*n* = 69) ensured a complementary, rich and strong array of data. Lastly, input from multiple researchers and a focus on multiple levels deepened the analysis, and thus were important strengths of this study. It should be noted that almost all participants were professionals [46] and volunteers [20], rather than people with dementia (1) and/or carers (2), despite our intention to recruit all relevant stakeholders. Our observations revealed that most of the DFI development is ‘organizationally led’ – that is, initiated by organizations with strong connections with the municipalities. Most professionals and volunteers from those organizations are still searching for ways to reach out to people with dementia and their carers, which could explain the low number of people with dementia and carers in our study. These limitations, are known from research about disengagement of people with dementia and their carers in development of DFCs [62–64]. Involvement of people with dementia and their carers during development and sustainment of DFI is lacking [62]. Instead, interest groups are involved as advocates [65]. People with dementia and carers are mainly questioned as ‘users’ of DFC about their priorities, experiences and outcomes of a DFC [9] but are not involved in the decision making process [63]. We mitigated the low number of people with dementia and carers in our study partly by conducting participating observations at DFIs so that fieldnotes about experiences of people with dementia and carers, could be incorporated in interviews and focus groups. People with dementia and their carers were invited to, and two carers took part in, the focus group to discuss the summary reports.

### Practical implications and future studies

The three MRPTs highlight the importance of building a support base, collaboration, and participation in DFIs by people with dementia and their carers during development and sustainment of DFIs. Our results should encourage stakeholders in practice to reflect on intermediate outcomes in order to monitor progress and follow up on all levels in the community. Moreover, our mechanisms provide more depth in this process by explaining the importance of a positive narrative and assets, and how events and intermediate outcomes on one level affect other levels in the community. In practice, such information is important in managing the process and understanding how and why things occur. Our results give a deeper insight in the process and succesfactors and can be used next to, for example, toolboxes that offer practical material [49] or describe planning during implementation of DFis [12]. Moreover, our results explain what important contextual conditions are during such a process and why – that is, which mechanisms arise and to which intermediate outcomes they lead on possible different levels of the community. Such insights will improve overview and reinforce the grip of stakeholders on the process of development and sustainment of DFIs. Future studies will be needed to further test and refine our MRPTs in other cases and contexts. Special attention is needed for the involvement of people with dementia and their carers during development and sustainment of DFIs. Insights from their perspectives will improve the understanding of building a support base, collaboration and participation in DFIs. We recommend a new study that can deepen the understanding of the perspectives of stakeholders, including people with dementia and carers regarding their involvement in the development and sustainment of DFIs. Next, future studies should address how people with dementia and their carers could be involved during the process of development and sustainment of DFIs. This will improve the management of the process.

## Conclusions

This study examined which contextual factors and mechanisms affected the development and sustainment of DFIs in a Dutch DFC, and the outcomes they produced. Two main conclusions can be drawn from the study. First, the results provide evidence about the importance of building a support base, collaboration and participation in DFIs by people with dementia and their carers. The accompanying mechanisms underpin the process of development and sustainment of DFIs and the importance of a positive narrative. Second, our results clearly suggest the interdependence between multiple levels in the community and how they impact the development and sustainment of DFIs. The results of our study can support practices of reflecting on intermediate outcomes and possible ripple effects. Accordingly, they can help stakeholders monitor their process of development and sustainment of DFIs. Our MRPTs may be used to support reflections on the process of development and sustainment of DFIs in a theory-based way.

This study provides transparency about the development and sustainment of DFIs, and is a reference point for future studies in which these theories can be tested and refined.

## Supplementary Information

**Additional file 1.** Topic guide.

**Additional file 2.** Data extraction form.

**Additional file 3.** Ten outlines cross case synthesis.

## Data Availability

Data analysed during this study are included in the published article and supplementary information files, including topic guide and templates used for data extraction. Templates with data extraction per case are available from the corresponding author on reasonable request.

## Abbreviations

DFC: Dementia friendly community
DFI: Dementia friendly initiative
CMOc: Context-mechanism-outcome configuration
MRPT: Middle range program theory
