# Supplementary material for: What matters in development and sustainment of community dementia friendly initiatives and why? A realist multiple case study
Source: BMC Public Health. 2023 Feb 9;23:296. doi: 10.1186/s12889-023-15125-9 (PMC9909928; doi:10.1186/s12889-023-15125-9)
Supplement: Supplementary file 1 — Additional file 1. Topic guide. [file 12889_2023_15125_MOESM1_ESM.docx]

**MENTALITY Phase B: Interview guide – PROFESSIONALS/VOLUNTEERS**

Research question Package B

How do stakeholders of 5-6 best practices of dementia friendliness in local contexts of the Netherlands and the UK describe DF initiatives, and what do they feel are important factors in creating and sustaining successful DF initiatives?

Please note: Each case (best practice) is considered as a bounded system of stakeholders in their local context.

Preparation

Organisation:

- Book/choose a quiet room
- Book your audio recording equipment and bring it along with you
- Make sure each participant has 2 informed consent forms (one for the interviewer, one for the interviewee)
- Provide an interview guide, paper and a pen to make notes, if necessary

- A card with your contact details and a little present to thank the participants.

Regarding content:

- Read all available information concerning the initiative (s) from the participant’s community/area/neighbourhood, and

- If possible, formulate programme theories which you can discuss in more detail during the interview
- Analyse as much as possible the conditions for a successful DFI, thereby taking into account aspects that are fixed and aspects that are changeable.

- Try to take part in the DFI prior to the interview. This will give you an idea of the initiative/activity (apart from the documentation). These observations, though not essential, will provide useful input during the interview.

- Find out more about the data extraction of the literature from package A, as this will allow you to put forward a suitable ‘rival programme theory (or CMOc) during the latter part of the interview.

- Make sure you are well-informed about the definitions of Context, Mechanisms (resources and responses) and Outcomes.

Regarding interview techniques

- **Summarising** is a very important interview technique, because you give the participant a recap of what you have heard. But you have to bear in mind that the objective is not to come up with a *comprehensive* summary. For that reason, make sure your summary is concise and to the point. It is as if you and somebody else are watching a film at the same time but you are not in the same room together, and you tell or ask each other what you see or what you think of the images on the screen.

**- Keep asking questions** is also an essential interview technique, especially when it comes to uncovering the so-called ‘hidden’ factors and mechanisms that participants are not always aware of. Ask for examples or ask questions such as: ‘could you tell us a bit more about this please?’ Try to get as clear an answer as possible, allowing you to visualise it yourself, like when you are watching the film together in separate rooms, as mentioned above.

- **What do you think was the reason for that? Why do you think it works that way?** These are very important questions that you should feel free to ask repeatedly, since they shed light on the causal link (from the perspective of the participant). Other techniques that are suitable here to keep asking questions are: How did you deal with [certain incidents, setbacks and windfalls]? In what sense did [certain incidents] impact on the success of the dementia friendliness initiative? In what sense did [incidents] influence the outcomes?

- Read the interview questions thoroughly, as this will allow you to keep asking questions at the right moment. This will turn the interview into a more fluent conversation.

-You will find that with some questions 1 or more versions are mentioned after the /: you will then have to choose what you feel best suits the participant and yourself.

- Listen carefully and choose the terms that best suit the participant. For example: if the term ‘initiative’ is not clear enough, opt for ‘activity’ or the word the participants are using themselves. This will make the conversation easier.

- Create some space to reflect on things, make some tea, take a break.

Introduction

- Explain the following aspects to the participant:
  - Objective and length of the interview: as many open questions as possible in order to recount experiences; there are no wrong answers in this! Our aim is to have a ‘conversation’, not to check off items on a questionnaire.
  - Indicate that in part 2 of the interview we would like to hear their opinion on aspects of the literature regarding dementia friendliness initiatives and communities
  - Use of audio recording equipment
  - Privacy measures, anonymous processing of data, secure storage of data
- Ask consent to use the audio recording equipment and ask the participant to sign the Informed consent form.
- Start the audio recording equipment
- Record prior to each interview: **the date of the interview and the code of the participant and the community.**
- START THE INTERVIEW

*Thank you for finding the time today to tell us about dementia friendliness initiatives and their profits. I was told that a lot of initiatives have been organised in your neighbourhood/area/community and that you take an active part in this. This interview is part of a study into the success factors of dementia friendliness communities. Our aim is to exchange views on dementia friendliness initiatives, what is actually working out, for whom, under which circumstances and what are the profits. I hope it will be a conversation in which we will be able to exchange a lot of information about our points of view and our ideas in this.*

| ***Question*** | **Comment** |
| --- | --- |
| *What do you consider to be important with regard to dementia friendliness? What do you think it actually means?* | To explore, to start up. |
| *I was told that various initiatives have been set up in your community/area/neighbourhood to increase dementia friendliness; could you tell me to what extent you are involved in this and what it is you do?* | When multiple activities are mentioned: focus on the activities of the interviewee themselves. |
| *(please react on this by saying things like: thank you, that is very good to hear, I would like to talk a bit more about some of these activities, which activities in particular would you like to discuss in more detail?)* | When the interviewee is involved in multiple activities within a DFI: first ask them what they would like to talk about themselves. Keep in mind another activity that you would like to know more about. That way you can discuss two activities into more detail. |
| *Keep asking questions about one or any of the activities mentioned:* | |
| *How did you get involved/started in this?* | Stay focused on the issue, otherwise you run a risk of taking too long here. |
| *What did you want to achieve?* | This question is especially aimed at the initiators of the activities: ask about choices and rationale/background of the initiative, allowing you to have a clear picture of the logic and the components. |
| *What is it in this environment/area/surroundings that makes it possible that what you are doing is successful here, in terms of atmosphere, people, social interaction, buildings, environment, history, etc.?* | Ask about context: people, buildings, social interaction, atmosphere, physical and social environment. Try to get answers to questions about cooperation partners on the level of the neighbourhood, area and community. |
| *What makes this environment special/unique, enabling these activities to be so successful here? Could this initiative be successful somewhere else as well? Why (not)? If we were to set up this initiative somewhere else, would that be successful and why (or why not)?* | Background: what are the conditions for the success of the initiative? What are in this respect fixed conditions (for example a law) and what are conditions that can be changed or influenced? |
| *What do you consider to be the success factors of this initiative/activity?*  *What makes the [dementia friendliness initiative] so special/typical, causing this environment to be more dementia friendly?* | Be aware of the fact that for the most part you will be given contextual factors about the initiative, so make sure that it does not turn into a list and/or that it becomes too generic.  If you are told of negative aspects of the context, ask the interviewee: how did you (and others involved) deal with this? In what sense did this impact on the implementation of the DFI? What were the consequences for the participants? |
| *What do you think triggers these success factors: how do you think that is working? What effects does that [what the person is doing, the activities] have on others?*  *What important part do you have in bringing about this success? Why do you think it works that way? How do you think it works? What does it trigger in others?* | Keep asking questions about the mechanisms |
| *Can you describe what they are subsequently doing? How do they react to this, what do you observe in their behaviour? What do you think is the reason for that? Can you give an example of that?* | Keep asking questions about the mechanisms |
| *What does the local resident suffering from dementia notice from this? How does this make things more dementia friendly for him/her? Can you give an example of that?* | From mechanisms to outcomes |
| *What do you think has been changed by the [dementia friendliness initiative], making it more dementia friendly for local residents with dementia or their informal carers? Do you also notice other effects? What is the reason for that, do you think? Why do you think it works that way? For whom does this work out, and for whom does it not, do you think? What is the reason for that?* | Outcomes |
| *Is there anything you would like to change in the [dementia friendliness initiative] in order to make it even better, and why?* | Questions to conclude (this initiative) |
| *What do you think we should know to be able to really understand how [the dementia friendliness initiative] has been successful here?* | Questions to conclude (this initiative).  Begin with asking about the next initiative when diving a bit deeper into this initiative. |
| *Who and what will be needed to keep up the initiative?* | Questions about implementation and sustainability.  Who: ask about the role of local residents or residents from the community.  What: ask about training, materials, money... |
| *So far we have discussed the initiatives and success factors in this environment. Research has also given us other ideas about dementia friendliness initiatives and what is working, for whom and why. So...(give an example from the literature of package A).* | Present the interviewee with outcomes from the literature, choose a ‘rival theory’: an example that is completely different from the initiative and the success factor discussed earlier on. |
| *Do you think that could work here as well? Why (not)? What concrete changes should be made in order to make it work?* |  |
| ***Conclusion***  *We would like to thank you very much for your time and for this conversation. Your answers will contribute to the development of dementia friendliness communities. It is possible that after this interview you might remember something that could be of value to us. We would like to know that very much! Some people contact us themselves, with others we arrange a time for us to phone them (just to be sure). Which would you prefer? What would be most convenient for you?*  *Do you know of anyone we should talk to, to hear their opinion on dementia friendliness*, its activities and success factors?*  *After we have spoken to several people we will conduct a final interview, in which we will give feedback on our outcomes. We would like to invite you to this interview [leave your card and ask/check the contact details you have of the person concerned].* | |
