# Supplementary material for: What matters in development and sustainment of community dementia friendly initiatives and why? A realist multiple case study
Source: BMC Public Health. 2023 Feb 9;23:296. doi: 10.1186/s12889-023-15125-9 (PMC9909928; doi:10.1186/s12889-023-15125-9)
Supplement: Supplementary file 3 — Additional file 3. Ten outlines cross case synthesis. [file 12889_2023_15125_MOESM3_ESM.docx]

**Additional file 3: Ten outlines after cross case synthesis**

| 1. **Support for a dementia friendly Initiative (DFI)** | | | | | |
| --- | --- | --- | --- | --- | --- |
|  | **Cases** | **Context** | **Mechanisms-resource** | **Mechanism-response** | **Outcomes** |
|  | Case A, B,C and D | People having expertise as a professional or by experience, who share their knowledge and insights during meetings.  Looking for potential partners and making yourself visible in/by your actions  Having history or experience in the public, health and social domain. Accessibility of the policy officers, volunteers and other partners, being visible and addressing each other.  Having attention for dementia and consequences, understanding of the needs and possibilities of partners in the community | Individual: sharing expertise, putting other people’s interest first.  Interpersonal: cultivating urgency, generating attention, goodwill.  Organisational: create opportunities to have a say, think along, encourage and complement each other, propagate vision of organization  Societal: recognition of both differences and similarities between populations reciprocity between different populations, understanding. | Individual: credibility, willingness.  Interpersonal: betrokkenheid, bereidwilligheid, verbondenheid, enthousiasme  Organisational: motivation, togetherness, sense of responsibility.  Societal: feeling urgency, willingness, interest, sense of responsibility | (Increase of) interest among community members,  greater awareness of needs among community members,  Motivation to contribute by professionals, volunteers and community members  Propose a joint approach by professionals, volunteers and community members  Understanding of the importance of the subject by by professionals, volunteers and community members |

| 1. **Public opinion-interaction** | | | | | |
| --- | --- | --- | --- | --- | --- |
|  | **Cases** | **Context** | **Mechanisms-resource** | **Mechanism-response** | **Outcomes** |
|  | Case A, B,C and D | People who are familiar with dementia, by knowledge or experience.  Personal and tailor made information about dementia and needs.  Information for different organizations and populations in the community.  Information by experts by experience.  Free access to information | Individual: recognizability and interest in information, feeling capable.  Interpersonal: learning to recognize symptoms of dementia, being used to different behavior.  Organisational: awareness of working methods, importance of alignment with populations  Societal: changing the image of people with dementia, recognizing experiences and pleasure, inspiration of ideas | Individual: reflection on one's own behavior, confidence in one's own actions, feeling of being important, feeling of being successful, understanding  Interpersonal: feeling more confident in company, understanding the other, feeling more positive towards the other.  Organisational: awareness and understanding  Societal: aroused interest, curiosity, better insight, increased insight into dementia | Awareness of consequences of dementia,  More positive image of own capacities,  Compassion,  Reflection on own behavior  Understanding of needs of populations.  being better informed about the impact of dementia and its consequences, decreasing anxiety |

| 1. **Set up of a Dementia friendly initiative (DFI)** | | | | | |
| --- | --- | --- | --- | --- | --- |
|  | **Cases** | **Context** | **Mechanisms-resource** | **Mechanism-response** | **Outcomes** |
|  | Case A, B,C and D | People with an affinity with the (people with) dementia and their carers.  diversity of partners in the community  professionals and volunteers presenting initial ideas to a familiar network,  The availability of driven initiator  Policy officers who are involved  Commitment and enthusiasm of volunteers and professionals. | Individual: insight into the value of a DFI, motivational communication, thinking about own contribution.  Interpersonal: become a partner, receive and give trust, be findable, take on one's own role.  Organisational: give recognition to others’ input, become a partner or co-owner. Sharing facilities, linking with own points of view and vision.  Societal: coordination of input and opinion between diverse populations and partners. propagating a can-do mentality | Individual: feel urgency for DFI, feel supported, experience willingness to take action.  Interpersonal: connection, trust, enthusiasm and appreciation.  Organisational: offer inspiration and stimulus, show willingness, offer support, work together.  Societal: feeling important, feeling taken seriously, giving and receiving recognition | Positive attitude towards people with dementia and carers,  Taking initiative for set up,  Willingness to take action,  Decisiveness,  Commitment towards partners to set up a DFI,  Cooperation with partners to set up a DFI |

| 1. **Set up a DFI-focus on perspective of people with dementia and their carers** | | | | | |
| --- | --- | --- | --- | --- | --- |
|  | **Cases** | **Context** | **Mechanisms-resource** | **Mechanism-response** | **Outcomes** |
|  | Case B,C and D | No recognition of suitable activities based on interests of people with dementia and their carers.  Evaluation of existing DFIs  information and facilities aimed at people with dementia  Ambition to become a DFC | Individual: Recognition of real interests and needs  Interpersonal: be informed and possibility to ask questions to the guide of DFIs  Organisational: attune to perspectives of people with dementia and their carers  Societal: special attention for dementia | Individual: feelings of urgency.  Interpersonal: feeling of being taken seriously  Organisational: connecting to needs  Societal: awareness of the importance of DFI | Inspiration for a DFI  Stimulated to set up a DFI  Incorporating needs and wishes in a DFI  Becoming an ambassador for a DFI |

| 1. **(retaining) people’s commitment to a DFI** | | | | | |
| --- | --- | --- | --- | --- | --- |
|  | **Cases** | **Context** | **Mechanisms-resource** | **Mechanism-response** | **Outcomes** |
|  | Case A, B,C and D | Linkage existing plans and possibilities.  Professionals and volunteers who feel in familiar territory  Making policy and making decisions together  Have contact with each other and recognize each other's expertise  Receive support and facilities from various organizations  Expressing ambition, paying attention to questions and objections | Individual: feeling positive energy, experiencing grip, feeling important for the DFI  Interpersonal: depend on each other, being involved, and taken seriously  Organisational: reshuffling tasks and roles if needed, providing overview and clarity of budget and manpower, offering and receiving support.  Societal: awareness of importance of DFI | Individual: feel involved, be motivated, experience willingness  Interpersonal: willingness, trust, togetherness and reciprocity.  Organisational: feeling supported, feeling important, equality, pleasure and motivation  Societal: feeling urgency, promoting solidarity, willingness | Commit yourself to a DFI and/or to others,  Stay active,  Experience pleasure and satisfaction,  Feel responsibility |

| 1. **Sustaining a DFI** | | | | | |
| --- | --- | --- | --- | --- | --- |
|  | **Cases** | **Context** | **Mechanisms-resource** | **Mechanism-response** | **Outcomes** |
|  | Case A, C, D. | Involvement of various organizations  (get to) know each other, both formally and informally  Give each other room for own actions,  Enjoyment  Acknowledgement of various levels in society needed for a DFI  Availability of evaluations,  Open access for community members at the DFI  Sufficient support from professionals  Sense of independence among citizens to take initiatives | Individual: feeling responsible, being informed  Interpersonal: share responsibility, acknowledgement of strengths, personal connection among each other  Organisational: clear roles, coordination of tasks and roles, knowing your vision on DFC and DFIs  Societal: hands on mentality | Individual: confidence, focus  Interpersonal: equality, solidarity, give each other something, proximity  Organisational: share responsibility, motivation, trust and appreciation  Societal: feeling responsible | Continue with own efforts  Improved collaboration,  Goal-oriented approach,  Sufficient availability of volunteers |

| 1. **Refer to a DFI** | | | | | |
| --- | --- | --- | --- | --- | --- |
|  | **Cases** | **Context** | **Mechanisms-resource** | **Mechanism-response** | **Outcomes** |
|  | Case B, C. | Contact with professionals for information  Urgency to connect with people with dementia and carers  Availability of information about DFI through the internet, face-to-face contact  Understanding needs of people with dementia and carers | Individual: curiosity, ask questions  Interpersonal: sharing experiences and information, knowing each other,  Organisational: sharing information and use information  Societal: use of PR | Individual: being informed,  Interpersonal: new insights, acknowledge value for people with dementia and their carers  Organisational: willingness to share, having an overview of facilities  Societal: - | Refer to own and other DFI,  Overview of DFIs and be able to make connections between DFIs,  Link people with dementia and carers to DFI, |

| 1. **Enlist professional care** | | | | | |
| --- | --- | --- | --- | --- | --- |
|  | **Cases** | **Context** | **Mechanisms-resource** | **Mechanism-response** | **Outcomes** |
|  | Case C, D. | Availability of professionals with expertise in dementia  Inability to answer questions  Acknowledgement of the need for other expertise and knowing where to find it  Public organisations such as housing corporation in network | Individual: being able to recognize dementia  Interpersonal: providing information, awareness of needs  Organisational: increase of knowledge.  Societal: - | Individual: have the courage to discuss own problems  Interpersonal: clarity and being informed, be more alert  Organisational: attention for people with dementia, feeling responsible, awareness of dementia,  Societal: | Refer to DFI  Refer to a case manager  Use of professional care  Organize, monitor and arrange adaptations |

| 1. **Participation of people with dementia and their carers** | | | | | |
| --- | --- | --- | --- | --- | --- |
|  | **Cases** | **Context** | **Mechanisms-resource** | **Mechanism-response** | **Outcomes** |
|  | Case A,B,C,D | Need for information and/or activities  Knowing participants of a DFI or volunteers/professionals  Personal approach  Avoiding dementia in announcements of location | Individual: having correct information, being able to make a choice based on interests.  Interpersonal: trust and commitment  Organisational: putting the DFI central, not medical conditions of participants.  Societal: open access | Individual: enthusiasm, feeling respected as a person  Interpersonal: willingness to offer help, feeling welcome  Organisational: inclusive approach, safety  Societal:- | Participation in a DFI by people with dementia and their carers  Make use of information and a supportive environment  Return of participants |

| 1. **Participation of community members** | | | | | |
| --- | --- | --- | --- | --- | --- |
|  | **Cases** | **Context** | **Mechanisms-resource** | **Mechanism-response** | **Outcomes** |
|  | Case A,B,C,D | Permanent location and guides  Knowing participants and guides  Availabilities of facilities  Appealing name of the activity  Open to everyone  Neutral location i.e. no care environment | Individual: familiarity with location clarity  Interpersonal: commitment, trust,  Organisational: open access  Societal: - | Individual: feeling welcome, accessible  Interpersonal: feeling stimulated feeling of belonging.  Organisational: inclusive approach, safety  Societal:- | Participation in the activity  Doing activities together |
